# Relationship between different physiological processes of Tomato seedlings exposed to acid mine water Uncovered using correlation analysis

**DOI:** 10.1016/j.heliyon.2023.e18975

**Published:** 2023-08-09

**Authors:** Udoka Vitus Ogugua, Sheku Alfred Kanu, Khayalethu Ntushelo

**Affiliations:** a Department of Agriculture and Animal Health, University of South Africa, Private Bag X6, Florida, 1710, South Africa; b Department of Crop Science, Njala University, Njala, Sierra Leone

**Keywords:** Tomato, Correlation, Germination indices, Heavy metals, Physiological parameters

## Abstract

This study was conducted to assess the correlation between growth response, phytoaccumulation factor of different tissues, and elemental composition in tomato seedlings exposed to acid mine water (AMW). In pairwise correlation determinations values of plant height, stem diameter, seed germination indices (radicle length, final germination percentage (FGP), emergency rate index (ERI), vigour index (VI), germination percentage (G%) and germination rate index (GRI)) and the elemental compositions (Cd, Cr, Cu, Ni and Zn) in the different plant tissues, root (root accumulation factor = RAF), stem (stem translocation factor = STF) and leaves (leaf translocation factor = LTF) were selected for the relationship determinations. Pearson correlation coefficients were calculated and revealed the relationships between the paired parameters. The study concluded that the strongly correlated physiological parameters were jointly co-ordinated in tomato seedlings exposed to AMW.

## 1 Introduction

Tomatoes are extensively grown over the world as a source of essential nutrients and their antioxidant properties. They are the second most cultivated vegetable in South Africa after potatoes. South Africa is a water scarce country with a long gold and coal mining history. Population growth will increase food demand, which will result in increased tomato production, especially if climate change causes droughts and clean water scarcity. Like in most agricultural soils in the world, long-term use of phosphatic fertilizers, sewage sludge application, dust from smelters, and industrial waste, can all add some heavy metals (Cd, Cu, Zn, Ni, Co, Cr, Pb, and As) in agricultural ecosystems [1]. Furthermore, mining activity that causes water pollution due to acid mine drainage (AMD) can severely harm crop production. Acid mine drainage with high quantities of trace metals contaminates mainstream rivers and seeps in communities around the mines either exposing crops to acid mine water (AMW) or forcing desperate farming communities to irrigate with AMW. The mining sector is concerned about the formation of AMD and the discharge of dissolved heavy metals (HMs) [2,3]. AMW has negative impact on the soil, vegetation, and even animals because of the harmful HMs that are not biodegradable and can accumulate in living organisms. Plants require a wide range of micronutrients; although some such as Cu, Cl, B, Fe, Mn, Zn, Mo, and Ni are required in trace amounts, others, such as Cd, Cr, and Pb, are considered pollutants [4]. Plants acquire appropriate amounts of each micronutrient, which necessitates the establishment of a metal homeostasis network comprising mobilization, uptake and distribution throughout the plant, intracellular trafficking, and storage. Because certain important metal ions are redox-active, they are found as catalytically active cofactors in many metalloenzymes. In addition to catalysis, some metals, such as zinc, have a structural role in protein stability [5]. In general, mineral elements are involved in a variety of activities in plants, including charge balance maintenance, electron carriers, structural components, enzyme activation, and supplying osmoticum for turgor and growth [ [6,7]]. The homeostatic balance of the plant is crucial especially when the plant has to deal with stress, such as exposure to AMW. Several mechanisms have been reported on how plants generally cope with such stress and includes reduced heavy metal uptake, binding to phytochelatins/metallothioneins (phytoextraction) [8], sequestration of heavy metals into vacuoles (phytoaccumulation/bioremediation). Tomato, a highly regarded model plant, has been extensively researched, particularly in the context of HM bioremediation. The plant exhibits a propensity towards phytoextraction due to the presence of toxic alkaloid solanidine within its leaves and stems, which may deter herbivores from consuming the metal-concentrating plant [9]. However, little or no study has evaluated the growth of tomato under short-term irrigation with mine water to establish the relationship between growth parameters and the root, stem and leaf heavy metal accumulation factors of tomato seedlings. To ascertain the relationship between plant height, stem diameter, different tomato seed germination indices (radical length, final germination percentage (FGP), emergency rate index (ERI), vigour index (VI), germination percentage (G%), root accumulation factor (RAF) Cd, RAF Cr, RAF Cu, RAF Ni, RAF Zn, stem accumulation factor (SAF) Cd, SAF Cr, SAF Cu, SAF Ni, SAF Zn, leaf accumulation factor (LAF) Cd, LAF Cr, LAF Cu, LAF Ni and LAF Zn, correlation analysis (CA) was done in this study.

Correlation analysis has been widely used to explain links and relationships between phenomena. Pearson correlation analysis is a common technique for determining the strength of a linear relationship between two variables. A matrix is simply displayed in a table or graph in CA to show the relationship judging by the correlation coefficient. Regarding the link between the different physiological processes of tomato seedlings exposed to AMW, there is a paucity of knowledge. Hence, the study aimed to determine the relationship between tomato seedlings’ growth response, phytoaccumulation factor of different tomato tissues, and elemental compositions using CA to explain the various physiological dynamics of tomato seedlings growing in AMD environments. Tomato was chosen to be the subject of this study because it is consistently being produced to meet the huge demand around the world; the crop is considered to be highly nutritious and with medicinal properties. Therefore, producers and consumers are very interested in evaluating the health risks associated with tomato consumption and other vegetables grown in polluted soils or irrigated with acid mine water due to clean water scarcity and frequent occurrence of drought associated with climate change.

## 2 Materials and methods

Acid mine water was collected from a gold mine in Randfontein, Gauteng, South Africa, and Table 1 highlights its physicochemical characteristics. For the germination indices experiment, seeds of the tomato cultivar Heinz 1370 were imbibed, in between Whatman no 1 paper filters, with water in Petri dishes (30 seeds per dish) and incubated at 20–25 °C. In six Petri dishes, seeds were imbibed with AMW and in another six with distilled water giving a total of 12 Petri dishes each having 30 seeds (360 seeds) for incubation. After seven days of incubation, the following parameters were determined; radicle length, FGP, ERI, VI and G%. Emergent seedlings (30) growing in the glasshouse were exposed to AMW by direct irrigation with 400 mL of AMW every two days from day 21 until 30 day (Fig. 2). Elemental compositions (Cd, Cr, Cu, Ni and Zn) in the different plant tissues root (RAF), stem (STF) and leaves (LTF) were determined on samples sampled at day 30 after planting as described by Nemutanzhela et al. [10]. Pearson correlation coefficients were calculated as per Table 2.

**Table 1:** Physicochemical parameters and heavy metal content of tap water and acid mine water (n = 9) sampled. The physicochemical parameters of the tap water and acid mine water were measured on the day of water collection from a gold mine. EC-electrical conductivity, TDS-total dissolved solute, DO- Dissolved oxygen.

PHYSICOCHEMICAL PARAMETERS	*TAPWATER (mg/L)	**AMD WATER (mg/L)
pH	4.0	8.4
Temperature (°*C*)	21.42	29
EC (*μS/cm*)	3151.33	68.98
TDS (*mg/L*)	74.00	145.35
NO_3_ (*mg/L*)	7.29	3.17
DO (*mg/L*)	80.54	20.09
SO_4_ (*mg/L*)	244.55	18515.33
**Heavy Metal**		
Cd	0.01	0.18
Cr	0.04	5.87
Cu	0.12	0.95
Ni	0.04	10.42
Zn	0.92	55.47

**Table 2:** Correlation analysis of plant growth and the accumulation factors of the root (RAF), stem (STF) and leaf (LTF).

	Plant height	Stem diameter	Radicle length	FGP	ERI	VI	G%	GRI	RAF Cd	RAF Cr	RAF Cu	RAF Ni	RAF Zn	STF Cd	STF Cr	STF Cu	STF Ni	STF Zn	LTF Cd	LTF Cr	LTF Cu	LTF Ni	LTF Zn
Plant height	1.00	0.68**	0.60**	0.32**	0.10*	0.23**	0.50**	0.52**	−0.66**	−0.11*	0.10*	0.06**	−0.60*	−0.58*	0.40*	−0.12**	0.33*	−0.63**	−0.62**	−0.32*	−0.02*	0.41**	−0.66**
Stem diameter		1.00	0.03*	0.02*	0.15*	0.05*	0.01*	0.01*	−0.93***	−0.79	−0.66**	−0.64**	−0.99***	−0.80**	0.36*	−0.63**	−0.30*	−0.99**	−0.87**	−0.78***	−0.69**	−0.35**	−0.99***
Radicle length			1.00	0.91***	0.71	0.86***	0.99**	0.99**	0.53**	0.42*	0.11*	0.97***	0.58**	0.11*	0.56**	0.66**	0.58**	0.53**	0.07*	0.86***	0.58**	0.61***	0.74**
FGP				1.00	0.93***	0.99**	0.97**	0.96**	0.80**	0.71**	0.00*	0.79**	0.29*	0.00*	0.29**	0.37*	0.29*	0.25*	0.00*	0.61**	0.32*	0.32*	0.46**
ERI					1.00	0.97***	0.78**	0.78**	0.97***	0.92***	0.05*	0.53**	0.09*	0.05*	0.09*	0.14*	0.09*	0.06**	0.09*	0.33*	0.09*	0.10*	0.36**
VI						1.00	0.92**	0.91**	0.87***	0.79**	0.00*	0.71**	0.21*	0.00*	0.21*	0.28**	0.21*	0.17*	0.02*	0.51**	0.21*	0.23*	0.20*
G%							1.00	0.98***	0.63*	0.53*	0.06*	0.92**	0.48**	0.06*	0.48**	0.56**	0.48**	0.43**	0.02*	0.78***	0.48**	0.51**	0.64**
GRI								1.00	0.61*	0.51**	0.07*	0.93**	0.50**	0.07*	0.50**	0.58**	0.50**	0.44**	0.03*	0.79***	0.50**	0.53**	0.66**
RAF Cd									1.00	0.78**	0.63**	0.68**	0.93**	0.96**	−0.38*	0.79**	0.34*	0.91**	0.99**	0.90***	0.75***	0.37*	0.95***
RAF Cr										1.00	0.97	0.97**	0.83**	0.72**	−0.11*	0.84**	0.66**	0.81***	0.76**	0.91***	0.96***	0.85**	0.81**
RAF Cu											1.00	0.93**	0.72**	0.57**	−0.06*	0.80**	0.76**	0.70**	0.60**	0.81***	0.95***	0.93***	0.68**
RAF Ni												1.00	0.42**	0.65**	0.06*	0.82**	0.65**	0.66**	0.67**	0.89***	0.93***	0.93**	0.67**
RAF Zn													1.00	0.80**	−0.45*	0.72**	0.44*	0.99***	0.87**	0.79***	0.77**	0.42**	0.99***
STF Cd														1.00	−0.33*	0.83**	0.33*	0.77**	0.99***	−0.35**	0.82***	0.34*	0.84**
STF Cr															1.00	−0.43**	−0.48 **	−0.43**	0.92**	−0.07**	0.86***	0.45**	0.76**
STF Cu																1.00	0.79**	0.67**	0.73**	−0.29*	0.95***	0.83***	0.74*
STF Ni																	1.00	0.40*	0.36*	0.22*	0.67**	0.70**	0.39*
STF Zn																		1.00	0.83**	−0.41*	0.70**	0.37*	0.99***
LTF Cd																			1.00	0.92***	0.75**	0.37*	0.90**
LTF Cr																				1.00	0.87*	0.69**	0.81**
LTF Cu																					1.00	0.83***	0.74**
LTF Ni																						1.00	0.38*
LTF Zn																							1.00

Signiﬁcance is indicated by asterisks. *P < 0.05, **P < 0.01, ***P < 0.001.

Note: FGP (final germination percentage), ERI (emergency rate index), VI (vigour index), G% (germination percentage) and GRI (germination rate index); RAF (root accumulation factor); STF (stem translocation factor); LTF (leaf translocation factor); +.70 or higher (very strong positive); +0.40 to +0.69 (strong positive); +0.30 to +0.39 (moderate positive) +0.20 to +0.29 (weak positive); +0.01 to +0.19 (no correlation); 0 (zero correlation); −0.10 to −0.19 (no relationship); −0.20 to −0.29 (weak negative); −0.30 to −0.39 (moderate negative); −0.40 to −0.69 (strong negative); −0.70 or higher (very strong negative).

### 2.1 Correlation between measured experimental parameters

**Fig. 1:**
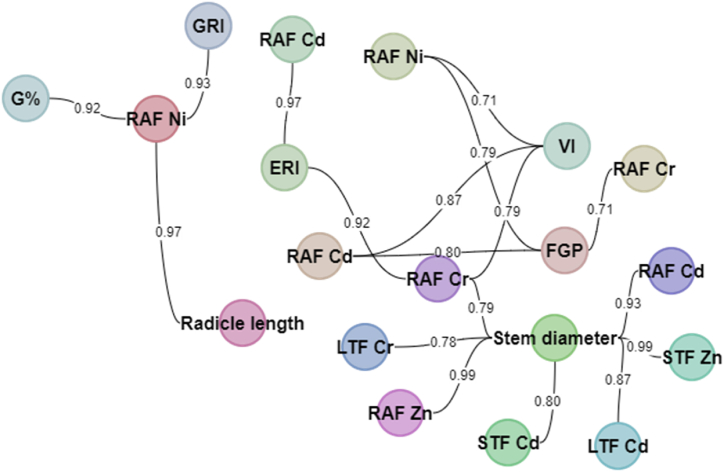
A nodal network showing positive correlation between the various measured parameters of seedling germination, growth, and heavy metal accumulation. The 18 nodes showed correlated parameters and the number between any two parameters is the correlation coefficient between those parameters. G%- germination percentage, RAF- root accumulation factor, GRI- germination rate index, RAF- root accumulation factor, ERI- emergency rate index, LTF- leaf translocation factor, STF- stem translocation factor, FGP- final germination percentage, VI- vigour index. The value between two parameters is the correlation coefficient between those parameters.

The relationship between different tomato seed germination indices, tomato different tissues, and elemental compositions was investigated using correlation analysis. Correlation between plant growth parameters (plant height and stem diameter), seed germination indices, and different plant organs (root, stem, and leaf) were calculated, the parameters which correlated were (plant height, stem diameter, radical length, FGP, ERI, VI, G%, RAF Cd, RAF Cr, RAF Cu, RAF Ni, RAF Zn, SAF Cd, SAF Cr, SAF Cu, SAF Ni, SAF Zn, LAF Cd, LAF Cr, LAF Cu, LAF Ni and LAF Zn) as shown on Table 2 and Fig. 1.

**Fig. 2:**
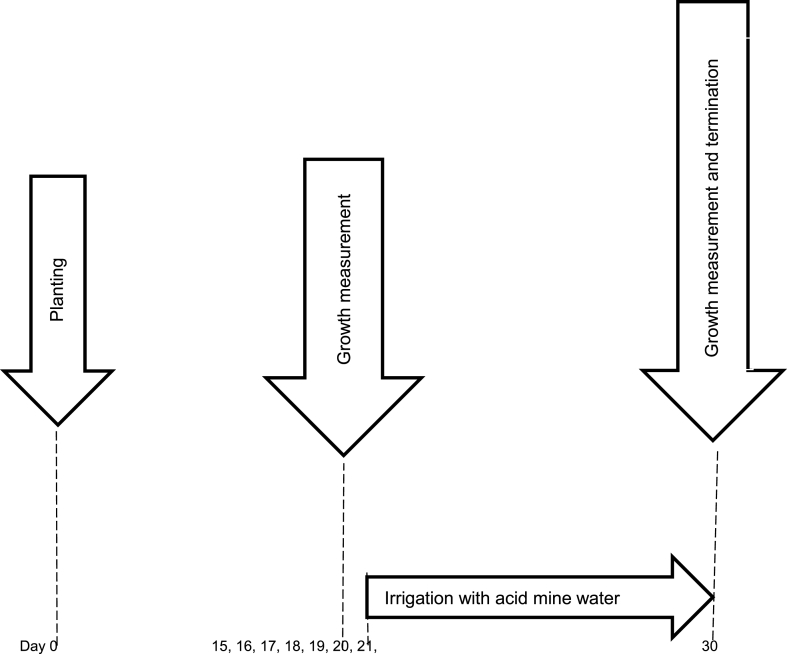
Timeline of the experiment. All parameters were measured at day 30 after planting.

We analyzed values of seed germination indices (radicle length, FGP, ERI, VI, G%, and GRI). Compositions of elements (Cd, Cr, Cu, Ni and Zn) in different organs of the plant (root, stem, leaf) were taken into consideration, and also, RAF, SAF, and LAF elements were used in the correlation analysis.

#### 2.1.1 Correlation matrix

The correlation between the plant growth parameters (plant height and stem diameter), seed germination indices, and elements found in different plant tissues (roots, stems and leaves) was analyzed by creating the correlation matrix and determining the levels of significance of the correlation coefficient using STATISTICA version 12 (StatSoft Inc., Tulsa, OK, USA). All pairwise correlation determinations are shown in Table 2.

The correlation coefficient (r) takes any value from the range −1 to +1. The sign minus or plus in front of the correlation coefficient indicates whether the variable is positively or negatively correlated. The absolute value of the coefficient shows the size of the correlation. Single regression analysis was used to assess the mineral availability of all analyzed parameters in this study.

For the strong positive correlations, the correlation diagrams were presented using the EzCorrGraph app [11], written in HTML5 and JavaScript language, the app generates the correlation diagram from a correlation matrix or a list of pairs with a simple and straightforward approach. The app is freely available on https://ezcorrgraph.firebaseapp.com/.

## 3 Result and discussion

Physicochemical analysis of acid mine water showed a higher pH, TDS, and SO_4_^−^ compared to tap water and the concentrations of Cd, Cr, Ni and Zn were also higher. This could be due to changes in acidic environment resulting in high levels of heavy metals availability to plants exposed to acid mine water. These findings are similar to results reported by Nemutanzhela et al. [10]. Generally, the correlation analysis result showed that correlations among plant growth parameters (plant height and stem diameter), seed germination indices, and different accumulation and translocation factors of HMs in plant organs (root, stem and leaf) were significant. The summary of the analysis (Table 2) showed the relationship between the plant growth parameters (plant height and stem diameter), seed germination indices (radicle length, FGP, ERI, VI, G%, GRI), HMs accumulation factors (RAF Cd, RAF Cr, RAF Cu, RAF Ni, RAF Zn; SAF Cd, SAF Cr, SAF Cu, SAF Ni, SAF Zn; LAF Cd, LAF Cr, LAF Cu, LAF Ni and LAF Zn) of the roots, stems and leaves of tomato seedlings grown in heavy metal laden acid mine water (Table 1).

Variations in the relationship between growth parameters (plant height, stem diameter, radicle length, FGP, ERI, VI, G%, GRI) and both stem and leaf for the measured HMs were observed. According to Table 2, most growth parameters showed a strong significant positive correlation with the translocation factor for germination indices and different HMs. However, there was a strong negative correlation between stem diameter and RAF Cd (−0.93), RAF Cr (−0.79), RAF Zn (−0.99), STF Cd (−0.80), STF Zn (−0.99), LTF Cd (−0.87), LTF Cr (−0.78), LTF Zn (−0.99). which have implications for the plant's ability to bioaccumulate HMs and enhance its movement to different organs for possible removal, possibly through leaf fall (Phytoextraction). The translocation of metal such as Zn through the leaves was negatively correlated (−0.99) to stem diameter. This implied that as the translocation of Zn to the leaves increases the stem diameter will decrease. In consonant with our findings, Al Khateeb and Al-Qwasemeh [12] observed a negative correlation of Zn at high concentration to the growth parameters (shoot length, number of roots and leaves, and fresh weight) of *Solanum nigrum*. As a consequence of the toxicity of the metal at the leaf level, crops may have fewer leaves, smaller stem diameters, and fewer buds due to altered biochemical and physiological activities [ [[13], [14], [15]]]. The transportation of the minerals occurs via the xylem in ionic form [16] with the help of organic acids [17]. The movement of HMs associated with organic acids from the roots to the aerial tissue will be affected, which may account for the decrease in the stem diameter and other growth parameters.

**Fig. 3:**
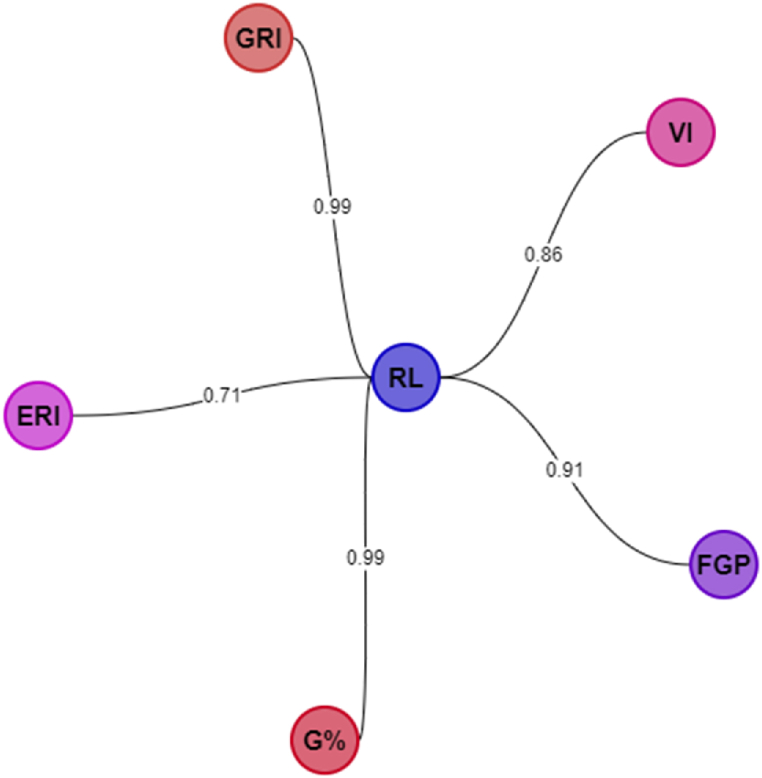
Radicle length versus FGP, ERI, VI, G% and GRI.

The strong positive correlation observed in radicle length versus FGP (+0.91), ERI (+0.71), VI (+0.86), G% (+0.99) and GRI (+0.99) (Fig. 3) shows that the germination indice is cardinal in germination processes for better seedling growth and development. The dearth of uniformity in seed germination may lead to poor seed establishment, thereby affecting the overall crop growth and yield [18]. This could be demonstrated by the activity of Sucrose/Raffinose Family Oligosaccharides (Suc/RFO). The Suc/RFO being a water-soluble sugar in plants are mobilized and support germination and seed vigour index. Suc/RFO is involved in protecting the cellular integrity systems when exposed to stress (such as pollution and salinity). Vandecasteele et al. [19] discovered that a high Suc/RFO ratio promotes germination and radicle proliferation. The relationship between sugars and germination/growth cannot be explained by their position as an easily accessible molecule; otherwise, increased Suc/RFO would be anticipated to improve germination and/or stimulate growth [19]. While previous research has shown that RFOs do not influence seed storability [[20], [21], [22]], there are conflicting findings in their studies on the role of RFO in seed vigour. On the one hand, Neus et al. [23] reported that low RFO soybean lines did not affect field emergence or other attributes such as seed yield and maturity when compared to a high RFO line. In contrast, suppression of α-galactosidase activity reduced germination during the imbibition of pea seeds, although a comparable investigation employing soybean seeds did not confirm the relationship between RFO breakdown and germination rate [24]. Although Suc/RFO was not determined in this study, the strong relationship of radicle length between G%; FGP; GRI and VI would suggest high activity of the Suc/RFO as stress (such as pollution and salinity) has been reported to increase the sugar concentration in tomato fruits [9].

**Fig. 4:**
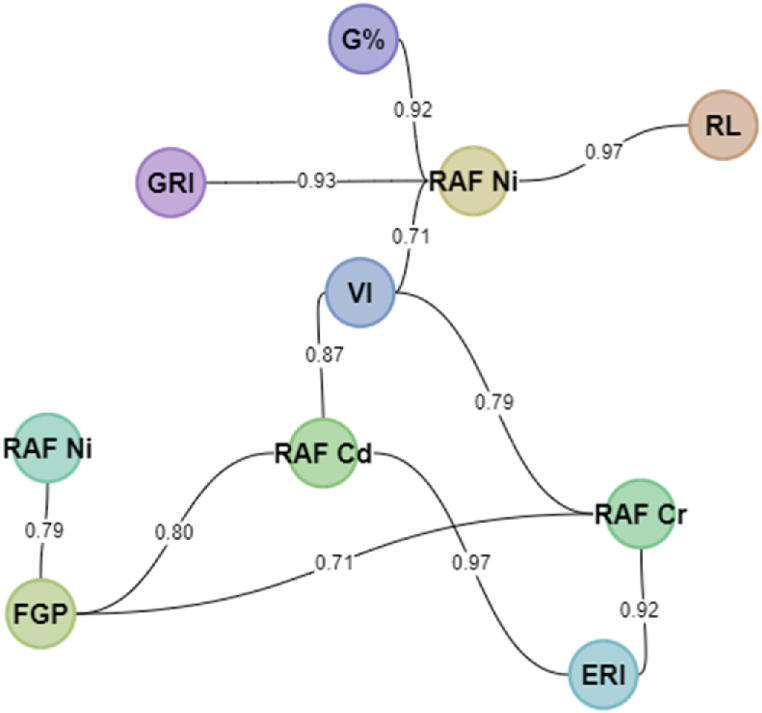
Radicle length (RL) versus RAF Ni, FGP versus RAF Cd, FGP versus RAF Cr, FGP versus RAF Ni, ERI versus RAF Cd, ERI versus RAF Cr, VI versus RAF Cd, VI versus RAF Cr, VI versus RAF Ni, G% versus RAF Ni, GRI versus RAF Ni.

Furthermore, the radicle length showed a strong correlation observation for the seed germination indices (radicle length, FGP, ERI, VI and GRI) and RAF (Cd, Cr and Zn): radicle length versus RAF Ni (0.97), FGP versus RAF Cd (0.80), FGP versus RAF Cr (0.71), FGP versus RAF Ni (0.79), ERI versus RAF Cd (0.97), ERI versus RAF Cr (0.92), VI versus RAF Cd (0.87), VI versus RAF Cr (0.79), VI versus RAF Ni (0.71), G% versus RAF Ni (0.92), GRI versus RAF Ni (0.93) (Fig. 4). Variation in positive correlation observed between the above-mentioned growth parameters and RAF for the associated metals indicated that their accumulation in plant roots is dependent on specific metal being absorbed [ [25,26]], of which, their translocation to other organs could trigger some biochemical process that may promote growth of radicals [17]. High biomass production and fast growth rates are the key characteristics of hyperaccumulators that enhance the maximum accumulation of metals [ [27,28]].

Accumulation factors provide an indication of the plants' ability to take up HMs and this ability is used to determine the plants’ phytoremediation ability and feasibility [29]. Plants which accumulate high quantities of HMs are therefore useful in operations to reclaim HM polluted environments. Accumulation of HMs has been recorded to have an adverse effect on the growth of crops especially plants that do not have the hyperaccumulating ability [15]. The correlation of the growth parameters of tomato and the HM accumulation factors of the root was done (Table 2). RAF Cd showed a strong positive correlation with RAF (Cr and Zn); as Cd, Cr, Cu, Ni and Zn increase in the root, the metals also increase thereby interfering with the movement and transportation of nutrients and water. As metal toxicity impaired movement of nutrients and water uptake [30] through the xylem. As a result of co-occurrence of HMs, tomato plants suffer from oxidative stress; lipid peroxidation, malondialdehyde presence and disruption of electron transfer chains. There is also an implication on the translocation of nutrients and water from underground parts to the aerial tissues of the plant. During seedling growth, heavy metal (Zn, Cd, Cu) contamination disrupted biochemical processes in the plant body by interfering with enzymes and chemical reactions resulting in growth inhibition [1].

**Fig. 5:**
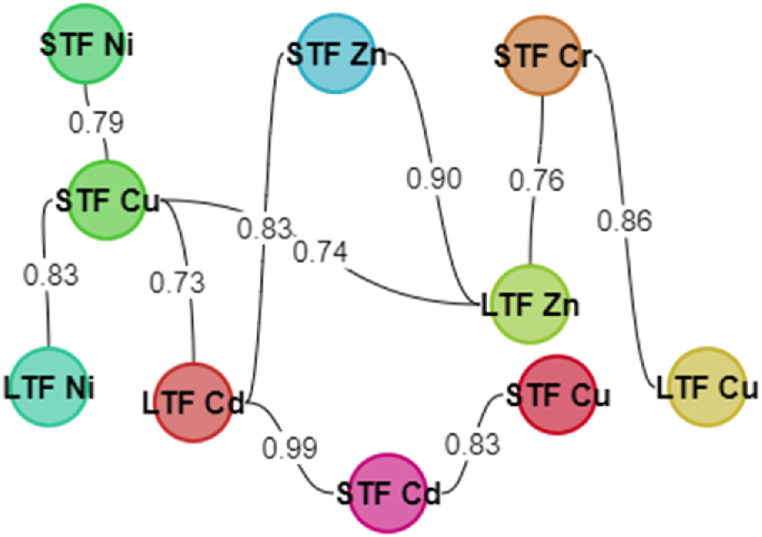
Stf Cd versus STF Cu, STF Cd versus STF Zn, STF Cd versus LTF Cd, STF Cd versus LTF Cu, STF Cd versus LTF Zn, STF Cr versus LTF Cd, STF Cr versus LTF Cu, STF Cr versus LTF Zn, STF Cu versus STF Ni (0.79), STF Cu versus LTF Cd, STF Cu versus LTF Cu, STF Cu versus LTF Ni, STF Cu versus LTF Zn, STF Ni versus LTF Ni, STF Zn versus LTF Cd, STF Zn versus STF Zn versus LTF Cu, STF Zn versus LTF Zn.

The ability of the plant to transport minerals from its roots to the aerial tissues is signified as the translocation factor; it is determined as the ratio of the mineral concentration in the plant's aerial tissues to the root concentration [ [31,32]]. They can transport certain minerals through cellular membranes as well as control the influx–efflux of metal translocation from roots to shoots [33]. The acropetal transfer to the shoot with the transpiration stream and subsequent redistribution in the phloem are critical for the distribution of nutrients and water uptake in the plant aerial tissues, beginning with root absorption and taking into the xylem and continuing in the phloem [5]. The correlation of the translocation factors (stem and leaves) (STF and LTF) from the RAF was shown (Table 2). Strong correlations were observed between the following parameters: STF Cd versus STF Cu (0.83), STF Cd versus STF Zn (0.79), STF Cd versus LTF Cd (0.99), STF Cd versus LTF Cu (0.82), STF Cd versus LTF Zn (0.84), STF Cr versus LTF Cd (0.92), STF Cr versus LTF Cu (0.86), STF Cr versus LTF Zn (0.76), STF Cu versus STF Ni (0.79), STF Cu versus LTF Cd (0.73), STF Cu versus LTF Cu (0.95), STF Cu versus LTF Ni (0.83), STF Cu versus LTF Zn (0.74), STF Ni versus LTF Ni (0.70), STF Zn versus LTF Cd (0.83), STF Zn versus STF Zn versus LTF Cu (0.70), STF Zn versus LTF Zn (0.90), LTF Cr versus LTF Cu (0.87), LTF Cr versus LTF Zn (0.81), LTF Cu versus LTF Ni (0.83), LTF Cu versus LTF Ni (0.74) (Fig. 5). Mwamba et al. [34] reported that Cd improved Cu absorption, particularly at low concentrations, but its own uptake was restricted by the presence of Cu. At either dose, the combined stress of these metals worsened plant growth inhibition and caused more oxidative damage than the individual metals. Only when Cu uptake was aided by Cd were the metals binding energy effects found. According to the study of Nan et al. [35], increasing Cd application increased Zn concentration in wheat and vice versa. McKenna et al. [36] discovered a complicated relationship between Zn and Cd accumulation in roots and leaves of lettuce and spinach. The environmental association of Cd and Zn, as well as their chemical similarity, can result in interactions between Cd and Zn during plant absorption, movement from roots to shoots, or accumulation in edible tissues. These interactions and their transfer show their synergistic effects in a plant's growth and development as increased Cd and Zn absorption in soil results in Cd or Zn build-up in plant tissues [37]. The existence of plasma membranes might explain the interaction and co-absorption of Cd and Zn [38]. Page and Feller [5] discovered that Ni is extremely phloem-mobile and is directed to the plant's aerial tissues. This was clear in our investigation since SAF Ni versus LTF Ni had a positive association (0.70), indicating that acropetal solutes may be transported from the xylem to the phloem. Nickel is a metal that travels through the phloem and is used to grow plant components [5].

### 3.1 Study Limitation

In this study, only seedlings growing in the glasshouse were exposed to AMW by direct irrigation with 400 mL of AMW every two days from day 21 until day 30 (short period). Therefore, data on growth and yield were not captured in this study and the fruits of tested tomato not analyzed for the presence of heavy metals. The study was conducted to decipher the correlation in the different physiological processes of tomato seedlings grown under greenhouse conditions.

## 4 Conclusion

The correlation analysis depicted a distinctive relationship between stem diameter and seed germination indices and also revealed antagonistic and synergistic interaction between the seed germination indices and elemental compositions in different tomato tissues (roots, leaves and stem). Utilization of Pearson's correlation shed light on the link between tomato seedlings growth response, phytoaccumulation factor of different tomato tissues, and elemental compositions. The future breeding strategies may consider the findings of this study especially in polluted environment in other to enhance tomato production.

## Declarations

### Author contribution statement

Sheku Alfred Kanu: Conceived and designed the experiments; Analyzed and interpreted the data; Wrote the paper.

Udoka Vitus Ogugua: Conceived and designed the experiments; Performed the experiments; Analyzed and interpreted the data; Wrote the paper.

Khayalethu Ntushelo: Conceived and designed the experiments; Analyzed and interpreted the data; Contributed reagents, materials, analysis tools or data; Wrote the paper.

### Data availability statement

Data included in article/supplementary material/referenced in article.

### Additional information

No additional information is available for this paper.

## Author's contributions

Conceptualization, U·V·O., S.A.K. and K·N; Investigation, U·V·O; Methodology, U·V·O; Resources, S.A.K. and K·N; Writing-original draft preparation, U·V·O; Writing—review and editing, U·V·O., S.A.K. and K.N. All authors have read and agreed to the published version of the manuscript.

## Funding

This research was fully funded by the 10.13039/501100001321National Research Foundation (10.13039/501100001321NRF) of South Africa, under grant number 121500.

## Institutional review board statement

Not applicable.

## Informed consent statement

Not applicable.

## Declaration of competing interest

I hereby confirm that there are no conflicts of interests for manuscript ref. **HELIYON-D-22**–**31419** “**Relationship between Different Physiological Processes of Tomato Seedlings Exposed to Acid Mine Water Uncovered Using Correlation Analysis**”.

All corrections suggested by reviewers have been addressed in the revise manuscript.
